# Dynamic cultivation of human stem cells under physiological conditions

**DOI:** 10.1186/1753-6561-9-S9-P68

**Published:** 2015-12-14

**Authors:** Dominik Egger, Markus Krammer, Jan Hansmann, Heike Walles, Cornelia Kasper

**Affiliations:** 1 Department Biotechnology, University of Natural Resources and Life Sciences, Vienna, 1070, Austria; 2 Chair Tissue Engineering & Regenerative Medicine, University Hospital Wuerzburg, 97080, Germany

## Background

The development of stable tissue-engineered autologous bone grafts in the field of regenerative medicine is still a challenge. Perfusion bioreactors not only provide continuous nutrition supply and waste removal, but are also suitable for the controlled application of mechanical forces like fluid shear stress. Mechanical loading is known to cause mechanotransductive effects like the induction of differentiation, resulting in enhanced deposition of extracellular matrix [1].

## Experimental approach

In our study, we determined the optimal flow rate for the osteogenic differentiation of human adipose-derived mesenchymal stem cells (MSC) by applying fluid shear stress that mimics the physiological environment normally experienced by bone progenitor cells in vivo. For this, we first analyzed the porosity of cell substrates with nanofocus-computed tomography as well as their specific permeability at different flow rates. To investigate the effect of controlled application of physiologic fluid shear stress a flow rate of 0.3 ml/min was used to cultivate MSC in a self-developed perfusion bioreactor. Cells were seeded on a three-dimensional macro-porous zirconium dioxide ceramic scaffold (0.3·106 cells/scaffold) and cultivated in standard growth medium (GM)or osteogenic differentiation medium (ODM) under normoxic (21% O2) or hypoxic (5% O2) conditions for a period of 21 days. After cultivation cell viability was examined using MTT assay. Furthermore DAPI staining was used to evaluate cell distribution. Glucose consumption and lactate production were monitored and histological stainings (calcein, alicarin red, Von Kossa) were used to evaluate osteogenic differentiation.

## Results

Flow rates between 0.3 - 5 ml/min result in fluid shear stress between 0.01 - 2.5 Pa which is in the range of physiologic shear stress bone progenitor cells are subjected to in vivo (0.3 - 3 Pa)[2]. Consequently a flow rate of 0.3 ml/min was used for perfusion culture. Cells cultivated on a 3D scaffold remained viable throughout the whole cultivation period. The viability of cells cultivated under perfusion was considerably higher (6-fold or higher) in comparison to static conditions throughout all conditions with the highest viability observed with cells in osteogenic medium with 5% O2 (Figure 1.1).Cell distribution was more homogenous under dynamic conditions. Although cells appeared to be denser in osteogenic medium than in standard medium no continuous cell layer was observed in any condition. Glucose consumption and lactate production were considerably higher under perfusion throughout all conditions (Figure 1.2). Cells cultivated at 5% O2 consumed more glucose and produced more lactate than under 21% O2. MSC are known to shift from oxidative phosphorylation to anaerobic glycolysis when cultivated under hypoxic conditions which causes a higher glucose consumption and lactate formation. During osteogenesis MSC rely more on oxidative phosphorylation resulting in a lower glucose consumption than during proliferation [3].Higher viability and elevated glucose metabolism seems to be a consequence of enhanced mass transfer due to perfusion cultivation. Matrix deposition (i.e. extracellular calcium and phosphate) was observed in both osteogenic and growth media. It was found to be strongest under dynamic conditions in osteogenic medium but even in standard growth medium devoid of osteoinductive supplements it was stronger than under static osteoinductive conditions. (Figure 1.3). Regarding the matrix deposition no difference was observed between 21% and 5% oxygen. Mechanical forces are known to have an impact on a variety of cellular processes such as the differentiation towards different lineages. Since the mineralization of the extracellular matrix is crucial for a tissue engineered construct mechanical forces need to be included into tissue engineering processes.

**Figure 1:**
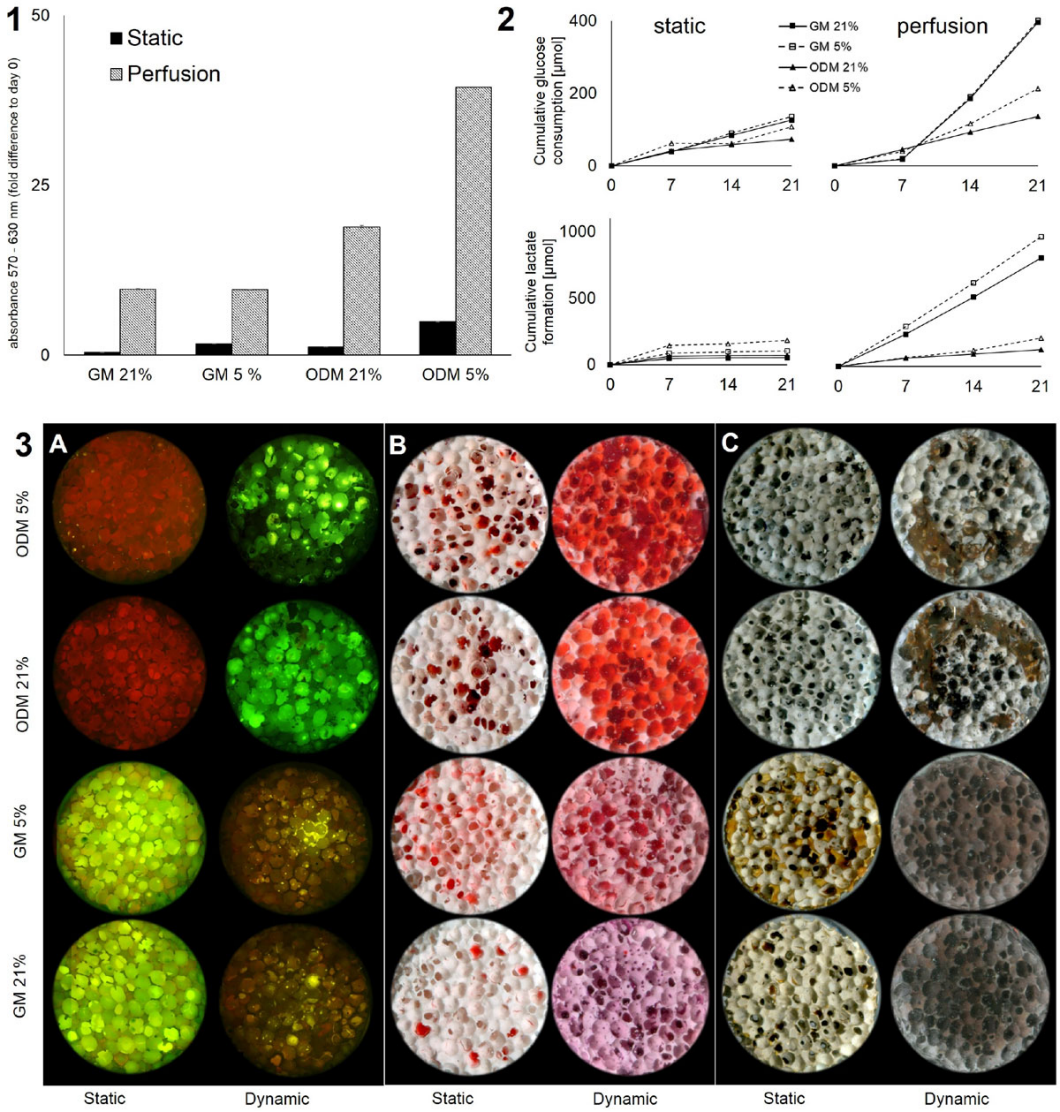
**MSC cultivated on a 3D ceramic scaffold for 21 days under static and dynamic perfusion conditions with 21% and 5% O_2_ in standard growth medium (GM) or osteogenic differentiation medium (ODM)**. (1) MTT-viability assay. (2) Glucose consumption and lactate formation. (3) Matrix deposition: (A) calcein staining for extracellular calcium, (B) alicarin red staining for extracellular calcium, (C) Von Kossa staining for extracellular phosphate.

## Conclusion

Physiologic fluid shear stress together with physiologic oxygen conditions (5% O2) lead to higher cell viability. Furthermore the glucose metabolism is elevated under perfusion due to enhanced mass transfer. The application of fluid shear stress results in a stronger differentiation regarding matrix deposition even in standard growth medium without any osteoinductive supplements. These results underline the positive effects of dynamic cultivation and physiologic oxygen concentration which together mimic in vivo conditions. Consequently other factors like medium composition should be adjusted to be more physiologic and taken into consideration during a tissue engineering process to ensure a physiologic tissue maturation.

## Acknowledgements

Part of this work was performed with financial support of the FFG BRIDGE project "3D Tissue".
